# Analysis of Paired Primary-Metastatic Hormone-Receptor Positive Breast Tumors (HRPBC) Uncovers Potential Novel Drivers of Hormonal Resistance

**DOI:** 10.1371/journal.pone.0155840

**Published:** 2016-05-19

**Authors:** Luis Manso, Silvana Mourón, Michael Tress, Gonzalo Gómez-López, Manuel Morente, Eva Ciruelos, Miriam Rubio-Camarillo, Jose Luis Rodriguez-Peralto, Miguel A. Pujana, David G. Pisano, Miguel Quintela-Fandino

**Affiliations:** 1 Medical Oncology Department, Hospital 12 de Octubre, Madrid, Spain; 2 Breast Cancer Clinical Research Unit, CNIO—Spanish National Cancer Research Center, Madrid, Spain; 3 Structural Computational Biology Group, CNIO—Spanish National Cancer Research Center, Madrid, Spain; 4 Bioinformatics Unit, CNIO—Spanish National Cancer Research Center, Madrid, Spain; 5 Biobank, CNIO—Spanish National Cancer Research Center, Madrid, Spain; 6 Pathology Department, Hospital 12 de Octubre, Madrid, Spain; 7 Translational Research Laboratory, Catalan Institute of Oncology, Bellvitge Institute for Biomedical Research, Barcelona, Spain; Florida International University, UNITED STATES

## Abstract

We sought to identify genetic variants associated with disease relapse and failure to hormonal treatment in hormone-receptor positive breast cancer (HRPBC). We analyzed a series of HRPBC with distant relapse, by sequencing pairs (n = 11) of tumors (primary and metastases) at >800X. Comparative genomic hybridization was performed as well. Top hits, based on the frequency of alteration and severity of the changes, were tested in the TCGA series. Genes determining the most parsimonious prognostic signature were studied for their functional role *in vitro*, by performing cell growth assays in hormonal-deprivation conditions, a setting that mimics treatment with aromatase inhibitors. Severe alterations were recurrently found in 18 genes in the pairs. However, only *MYC*, *DNAH5*, *CSFR1*, *EPHA7*, *ARID1B*, and *KMT2C* preserved an independent prognosis impact and/or showed a significantly different incidence of alterations between relapsed and non-relapsed cases in the TCGA series. The signature composed of *MYC*, *KMT2C*, and *EPHA7* best discriminated the clinical course, (overall survival 90,7 vs. 144,5 months; p = 0.0001). Having an alteration in any of the genes of the signature implied a hazard ratio of death of 3.25 (p<0.0001), and early relapse during the adjuvant hormonal treatment. The presence of the D348N mutation in *KMT2C* and/or the T666I mutation in the kinase domain of *EPHA7* conferred hormonal resistance *in vitro*. Novel inactivating mutations in *KMT2C* and *EPHA7*, which confer hormonal resistance, are linked to adverse clinical course in HRPBC.

## Introduction

Hormone receptor–positive breast cancer (HRPBC) is a heterogeneous disease, and a major therapeutic problem in this breast cancer subtype is the acquisition of hormonal resistance[1]. The positive results of everolimus[2] and palbociclib[3] in randomized phase III trials show that different mechanisms (i.e., MTOR activity or aberrant cell cycle control) account for this event. Many preclinical studies suggest as well that the acquisition of hormonal resistance is multifactorial[1]. Despite the success of the former therapies, it is clear that other relevant mechanisms are responsible for the acquisition of hormonal resistance, demonstrated by the fact that a percentage of patients is refractory to those therapeutic alternatives, and ultimately all the patients experience relapse[2, 3].

Next-generation sequencing (NGS) studies have helped to understand how different cancers evolve with time, specially under the selective pressure induced by exposure to different targeted agents. Campbell and colleagues pioneered the study of the evolution of cancers at the clonal level[4, 5]; however, in those studies the patients were not stratified specifically by therapies. More recently, different studies have shown how tumors acquire novel mutations[6] or expand specific clones [7] in response to specific therapies, by analyzing paired samples. Large-scale studies sequencing incident cases [5, 8–12], have provided invaluable information about the genomic landscape of breast cancer. However, the relatively low sequencing depth and the lack of paired-samples analysis complicate the detection of variants present in clones with small representation in the primary tumor (that may expand and account for disease relapse), or pinpointing genes accounting for specific clinical outcomes or features, respectively. In contrast, smaller studies focusing in paired samples (primary tumors plus their metastasis), have discovered novel variants associated to hormonal failure, such as the acquired mutations in the *ESR1* gene[13–15].

Besides *TP53*, *PIK3CA*, and *PTEN*, most genes are reported as being mutated in <10% of incident cases [4, 5, 8–10, 12, 16, 17]. Although several associations between the mutations in those genes and disease progression while in hormonal treatment have been observed, the definitive prognostic/predictive value is still unclear[18–22]. In addition, the metastatic phenotype might be acquired through the independent evolution of clones not present in the primary lesions, rather than being caused by mutations present at a low level, and thus be undetectable in a comparison of different primary cases. Given the heterogeneity of HRPBC, it is possible that genes other than *TP53*, *PIK3CA*, and *PTEN* that are mutated in small percentage of cases account for cases of failure to hormonal treatment and disease relapse.

Thus, we sought to detect genetic alterations involved in an adverse disease course and hormonal resistance by studying a set of HRPBC where all the cases experienced distant relapse. We analyzed the primary tumors paired with their metastases, by sequencing them at ultra-high depth and performing comparative genomic hybridization (CGH). We then tested candidate genes in an independent series and conducted *in vitro* studies of those that showed external prognostic value, pinpointing novel candidate genes that potentially account for hormone resistance and long term relapse of HRPBC.

## Materials and Methods

### Study population and ethics board

Women with a histologic diagnosis of HRPBC, for whom tissue from a distant metastasis and full medical records were available, were eligible. Patients with synchronous metastases were excluded. The study protocol was approved by the Institutional Review Board of Hospital 12 de Octubre ("Comité Ético de Investigación Clínica—Hospital 12 de Octubre", Madrid, Spain) (Study code: CNIO-BR-004), and conducted according to the principles expressed in the Declaration of Helsinki. This review board waived the need for consent since all the samples belonged to patients diagnosed of cancer before 2007. According to the Royal Act in Biomedical Research in force in Spain since 2007 (Royal Act 14/2007, July 3^rd^), the retrospective collection of archival samples belonging to patients diagnosed before 2007 do not require individual signed informed consent.

### Tissue processing, DNA sequencing, and comparative genomic hybridization

Areas with >90% epithelial tumor content from formalin-fixed, paraffin-embedded tissue sections were laser-capture macrodissected.

A custom panel covering the coding DNA sequence of the 106 genes that are known to be altered in at least 1% of the HRPBC cases[4, 5, 8–10, 16, 17] was designed with SureSelect technology, and an Illumina HiSeq2000 device was used. The depth was set to a minimum of 500X to enable studying very low minor allele fractions (MAFs) and their changes. Copy number alterations (CNAs) were studied by comparative genomic hybridization (CGH) using a Human Whole Genome 8x60k oligonucleotide array-CGH (Agilent Technologies), following ULS labeling protocol, to query the 101 regions gained or lost (CNAs) in at least 1% of HRPBC cases [4, 5, 8–10, 16, 17]. Thus, more than 99% of the known genetic alterations in HRPBC were assessed (S1 Table). Of note, ESR1 was not included in the panel, since by the time this study was designed no mutations had been detected in this gene despite having been sequenced in several series of primary tumors [4, 5, 8–10, 16, 17]. The discovery of ESR1 activating mutations came almost one year later with whole-exome sequencing studies of metastastic tumors[13–15].

Microarray data were extracted and visualized using Feature Extraction software v10.7 and Agilent Genomic Workbench v5.0 (Agilent Technologies). CNA regions were detected using the ADM-2 (set as 6) statistic provided by DNA Analytics, with a minimum number of 10 consecutive probes. The segmentation process was carried out using the dnacopy Bioconductor package [23]. Bioconductor´s CGHcall package was employed for determining the step, and CGHregions and CGHtest packages[24] were used to estimate genomic regions and false discovery rate, respectively.

Microarray and sequencing data have been deposited in GEO and SRA, under the following accession number: GSE79446 and SRP071834, respectively.

### Sequence alignment, variant calling, functional annotation and heatmap generation

Raw FASTQ sequence files were aligned using BWA 0.7.5 software [25]. Alignment metric generation and duplicate sequence marking were performed with Picard 1.107 (http://broadinstitute.github.io/picard). Single nucleotide variations were determined for MAFs > 1% with VarScan2 [26]. Variant annotation was performed using PROVEAN web server tool [27], which implements both PROVEAN and SIFT functional severity predictors. Variants mapping to the same genomic coordinates as known polymorphisms (annotated with a dbSNP ID) were discarded. Severe impact variants were retained for further analyses if they were simultaneously predicted as *Deleterious* by PROVEAN (cutoff = −2.5) and as *Damaging* by SIFT (cutoff = 0.05). The heatmap was produced by using the average clustering method on Binary Euclidean´s distances computed over binary data matrices (i.e., variant present or absent in the sample, with assigned values of 1 and 0, respectively).

### Generation of hormone-resistant clones and *in vitro* experiments

Eleven hormone-receptor positive breast cancer cell lines (KPL1, EVSAT, ZR75-1, CAMA1, HCC1428, HCC1500, MCF7, T47D, MDA-MB175-VII, BT483, and MDA-MB-415) were acquired from the ATCC. Cell-line authentication was done periodically every six months, utilizing short-tandem repeat profiling, by re-sending cell-line pellets to the ATCC. Cell line clones resistant to estrogen deprivation were generated in an *in vitro* model analogous to acquisition of resistance to aromatase inhibitors, as described by Martin *et al* [28], Briefly, the method consisted of weekly passage and culture of cells in medium containing 10% dextran charcoal-stripped (DCC) fetal bovine serum (FBS) instead of full FBS, which removes steroids. The medium was changed every 2–3 days. The cells underwent a three-phase process: quiescent long-term estrogen deprivation (LTED-Q), followed by a phase of hypersensitivity to estrogens (LTED-H), and finally an apparent estrogen-independent phase (LTED-I). This process usually lasts more than a year; several HRPBC cell lines cannot withstand the process and die during the first passage(s) or remain quiescent.

Cytotoxicity assays were performed in triplicate with either parental cell lines or LTED cultures, which recovered for at least two passages in full medium and were then plated and allowed to grow in 96-well plates (10000 cells per well) for 5 days with full medium or DCC medium. Surviving cell were then estimated using the luciferase reaction to measure the amount of ATP from viable cells (CellTiter-Glo® Luminescent Cell Viability Assay (Promega), following the manufacturer’s instructions.

An *EPHA7* wildtype construct were obtained from Plasmid Collection (Harvard Medical School, Clone ID HsCD00416965) and the point mutation T666I was introduced using the QuikChange Lightning Site-Directed Mutagenesis Kit (Agilent Technologies), following manufacturer´s instructions. Plasmids were transiently transfected with Lipofectamine® 2000 (Life Technologies). Stable cell lines were generated by infection of a TRIPZ lentiviral vector carrying an inducible shRNAmir targeting *EPHA7* gene (Open Biosystems). Expression of *EPHA7* shRNAmir was induced with 1 μg/ml doxyxyclin (Dox) for 5 days. EPHA7 mRNA levels were measured through real-time PCR in a 7500 Fast real-Time PCR System (Applied Biosystems) apparatus.

### Statistical analysis

The individual prognostic value of each gene in the TCGA series was calculated with the Kaplan-Meier method and the Log-Rank test. The statistical significance for a differential incidence in gene alterations in the alive versus death patients in the TCGA series was calculated with a Z-test, adjusted by multiple comparisons. We also tested whether the presence of alterations in the genes under study was individually associated with disease relapse by using a Fisher´s exact test. The overall survival comparisons between the patients with and without the three-gene signature were performed with the Kaplan-Meier method and the Log-Rank test. The hazard ratio of death attributable to the gene-signature was calculated with the Cox´s proportional hazards method. This calculation was performed twice, performing an univariate and a multivariate model. In the second cases, the risk of death conferred by the gene signature was adjusted by age, T- and N-stage. All tests were performed with the SPSS Statistics V.19.0 software.

## Results

### Discovery set summary: selection of candidates for external testing

Patients' demographic data are provided in Table 1. The mean sequencing coverage was above 800× for all samples. A total of 1071 unique base-pair variations from the reference genome were identified. As predicted by the PROVEAN/SIFT tools, 39 of the variations could have severe functional consequences in 27 distinct genes. Table 1 shows by case which genes were affected by severe alterations, and whether they were present in the primary/metastatic/both lesions. We found the K178N nucleotide change in *NCOR1* in all cases. Another frequently mutated gene was *KMT2C*, which encodes for the histone lysine-specific methyl transferase 2C (a tumor suppressor in acute myeloid leukemia[29]). The D348N mutation was present in four pairs. According to the CGH arrays data, three regions (encoding, respectively, KDM5C, KDM6A and FOXO4) were gained in all but one pair (S2 Table).

**Table 1:** Clinical Characteristics and Detected Deleterious Mutations in Each Patient Pair.

Pair	Metastatic to	Ki-67	TTR*	Stage**	Sample site	AFF2^+^	ARID1B	CAMK1D	CASP8	CDH1	CSF1R	DDR2	DNAH3	DNAH5	EPHA7	FOXA1	GATA3	KDM5B	KMT2C	KMT2D	LAMA4	LAMB3	MAP3K12	MAP4K4	MYB	MYC	MYH9	MYO3A	NCOR1	RASGRF1	TP53	USH2A
A	Bone	12%	11,8	T2N1	Primary														**1**										**1**			
					Metastasis														**1**										**1**			
C	Peritoneum	5%	3,9	T2N1	Primary			**1**								**1**	**1**				**1**							**1**	**1**			
					Metastasis																**1**								**1**			
D	Peritoneum	12%	4	T2N1	Primary		**1**																**1**		**1**				**1**			
					Metastasis		**1**																		**1**				**1**			
E	Peritoneum	6%	7,3	T2N1	Primary					**1**									**1**										**1**			
					Metastasis					**1**							**2**												**1**			
I	Peritoneum	3%	15,1	T3N1	Primary																								**1**			
					Metastasis																								**1**			
J	Peritoneum	13%	4,1	T2N2	Primary																					**1**			**1**			
					Metastasis																					**1**			**1**			
K	Lung	6%	10,6	T2N1	Primary														**1**										**1**		**1**	
					Metastasis														**1**										**1**		**1**	
L	Peritoneum	30%	4,8	T2N2	Primary														**1**										**1**			
					Metastasis														**1**								**1**		**1**		**1**	
M	Bone	30%	1,8	T3N0	Primary																								**1**			
					Metastasis	**1**					**2**	**1**	**1**	***4***				**1**		**2**	**1**			**1**				**1**	**1**	**1**		**1**
O	Bone	11%	3,3	T2N0	Primary										**1**							**1**							**1**			
					Metastasis				**1**						**1**							**1**							**1**			
T	Bone	5%	4,1	T2N2	Primary														**2**										**1**			
					Metastasis														**1**										**1**			

*TTR: Time to relapse: time since the diagnosis of breast cancer until the diagnosis of metastatic relapse

**Stage: tumor and nodal stage at diagnosis

For each tumor, each numbered cell corresponded to one (or more) deleterious mutations detected either in the primary or metastatic lesion for each of the depicted genes of interest.

Genetic variants implicated in an aggressive disease course may be detected by studying those with increased clonal representation in the metastatic lesion because it relates to an evolutionary advantage. The MAF expansion for the 39 variants is shown in Table 2. With the exception of case M, few variants had a representation that changed by more than 10% in more than one pair. Virtually all the CNAs remained unchanged (S2 Table).

**Table 2:** Allelic Expansion of Variants Causing Severe Functional Protein Alterations from the Primary to the Metastatic Lesions.

Pair ID	Gene	Primary tumor MAF	Metastatic tumor MAF	MAF expansion
***A***	*KMT2C*	0.49	0.37	−0.12
	*NCOR1*	0.48	0.52	0.03
***C***	*LAMB4*	0.41	0.51	0.1
	*CAMKD1*	0.26	0.05	−0.21
	*MYO3A*	0.12	0.04	−0.08
	*GATA3*	0.13	0.03	−0.1
	*FOXA1*	0.2	0.05	−0.15
	*NCOR1*	0.54	0.49	−0.05
***D***	*MAP3K12*	0.26	0	−0.26
	*MYB*	0.52	0.49	−0.02
	*ARID1A*	0.35	0.53	0.16
	*NCOR1*	0.51	0.54	0.03
***E***	*CDH1*	0.20	0.56	0.36
	*KMT2C*	0.11	0.10	−0.01
	GATA3*	0	0.41	0.41
	*NCOR1*	0.42	0.36	−0.06
***I***	*NCOR1*	0.42	0.42	0
***J***	*MYC*	0.48	0.49	0.01
	*NCOR1*	0.57	0.42	−0.15
***K***	*KMT2C*	0.11	0.11	0
	*NCOR1*	0.42	0.42	0
	*TP53*	0.33	0.24	−0.09
***L***	*KMT2C*	0.11	0.11	0
	*NCOR1*	0.55	0.52	−0.03
	*TP53*	0	0.15	0.15
	*MYH9*	0.07	0.19	0.12
***M***	*DDR2*	0	0.37	0.37
	*KDM5B*	0	0.11	0.11
	*USH2A*	0	0.39	0.39
	*MYO3A*	0	0.38	0.38
	KMT2D**	0	0.48	0.48
	*RASGRF1*	0	0.53	0.53
	*DNAH3*	0	0.11	0.11
	*NCOR1*	0.38	0.53	0.15
	*MAP4K*	0	0.43	0.43
	DNAH5***	0	0.54	0.54
	*CSF1R*	0	0.85	0.85
	*LAMA4*	0	0.46	0.46
	*AFF2*	0	0.91	0.91
***O***	*LAMB3*	0.55	0.65	0.1
	*EPHA7*	0.27	0.47	0.2
	*NCOR1*	0.28	0.44	0.16
	*CASP8*	0	0.46	0.46
***T***	*KMT2C*	0.13	0.23	0.1
	*NCOR1*	0.37	0.42	0.05

"Primary tumor MAF": fraction of reads from 0 to 1 in which the mutation was detected in the DNA from the primary tumor. "Metastatic tumor MAF": same, for the metastatic tumor. "MAF expansion": increase or decrease in the fraction of representation of the variant allele in the metastatic versus the primary lesion.

* Two mutations were detected for *GATA3* in E; the second expanded from 0 to 0.27.

** Two mutations were detected for *KMT2D* in M; the second expanded from 0 to 0.43.

*** Four mutations were detected for *DNAH5* in M; the other three expanded from 0 to ∼0.4.

**Fig 1:**
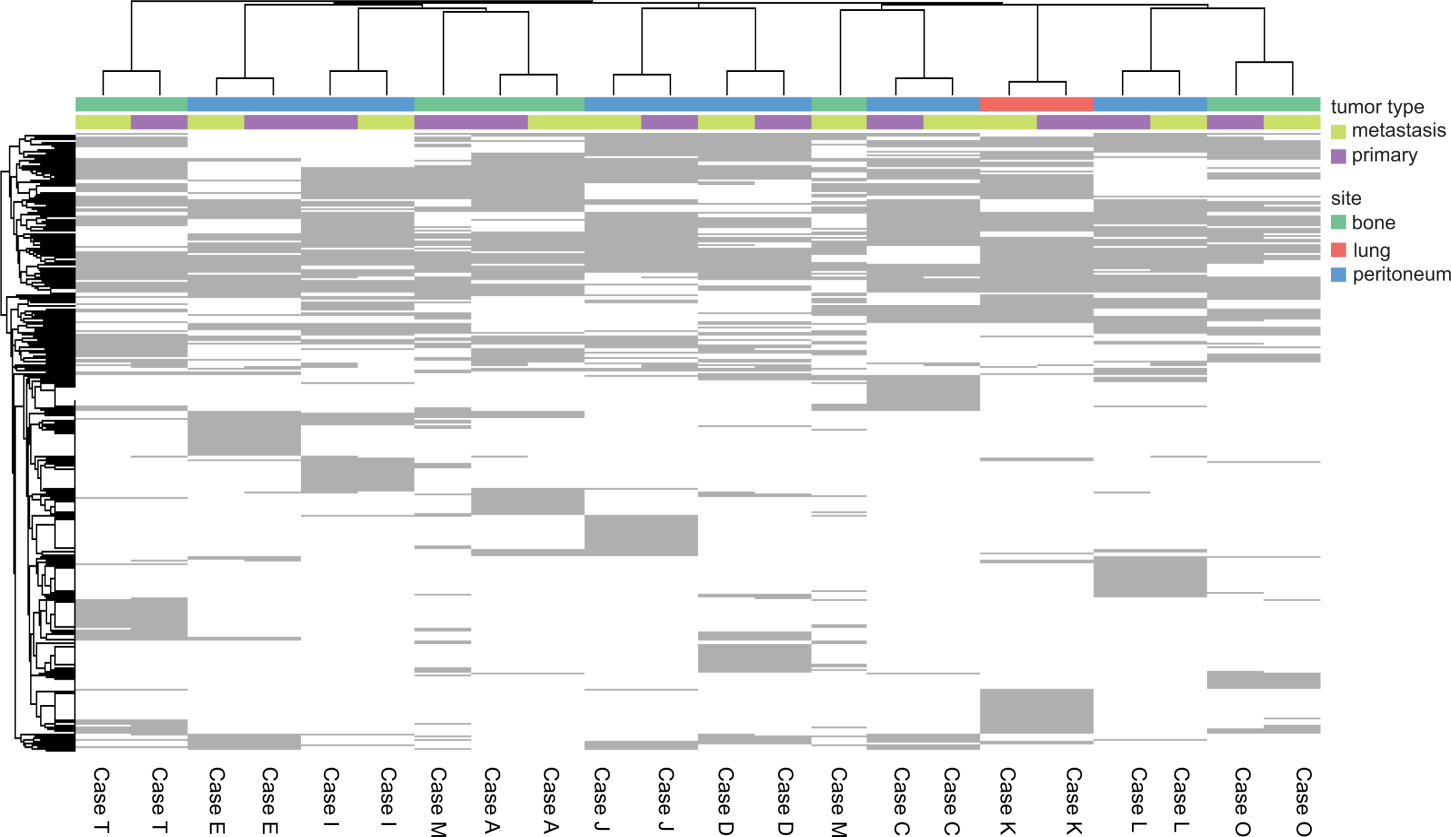
Variant call heat map. Each of the 1071 unique variants identified are depicted in the heatmap as "present" (numbered cell) or "absent" (empty) in each primary or metastatic tumor. With the exception of pair M, each tumor was more similar to its pair than to any other case, regardless of being primary (pink-colored cases) or metastatic (yellow-colored cases), or metastatizing into the same organs or not (green: bone; red: lung; blue: peritoneum).

Globally, the variants were stable from the primary to the metastatic lesions (>99.9% of the base pairs of the >500 kilobase region sequenced were conserved). An unsupervised clustering showed that each case was more similar to its paired metastatic lesion than to any other case (except from case M, Fig 1). Whether one or more of the 1071 variants differed between the metastatic and primary lesions in each pair is shown in S3 Table (by genes and pairs).

Since our series was constituted exclusively by cases that experienced distant relapse, we aimed to explore whether any of those genes in where we found potential severe alterations (predicted by PROVEAN or SIFT) was recurrently affected in relapsing cases in a larger external series, in order to generate potential predictive signatures. From those 27 genes we excluded *AFF2*, *CAMK1D*, *CASP8*, *DDR2*, *DNAH3*, *FOXA1*, *KDM5B*, *MAP3K12*, *MAP4K4*, *MYH9*, *RASGRF1* and *USH2A* because of being mutated in only one lesion from one pair each. We added to the remainder 15 *KDM6A*, *FOXO4* and *KDM5C*, since they were amplified in many pairs of our series. Finally, although we did not find a high percentage of pairs with mutations in either *PIK3CA* or *PTEN*, we included them in the panel of genes that we used for generating parsimonious predictive signatures, since most series report a high frequency of mutations in those genes in incident cases[5, 8–10, 16]. Thus, the panel of genes that we tested externally was: *TP53*, *MYC*, *CDH1*, *LAMB3*, *KMT2C*, *NCOR1*, *GATA3*, *LAMA4*, *MYB*, *DNAH5*, *ARID1B*, *KMT2D*, *MYO3A*, *EPHA7*, *KDM6A*, *FOXO4*, *KDM5C*, *CSF1R*, *PIK3CA* and *PTEN*.

### External testing: three-, four-, five- and six-gene signatures point towards KMT2C and EPHA7 as potential drivers of hormonal resistance because of their prognostic impact

**Fig 2:**
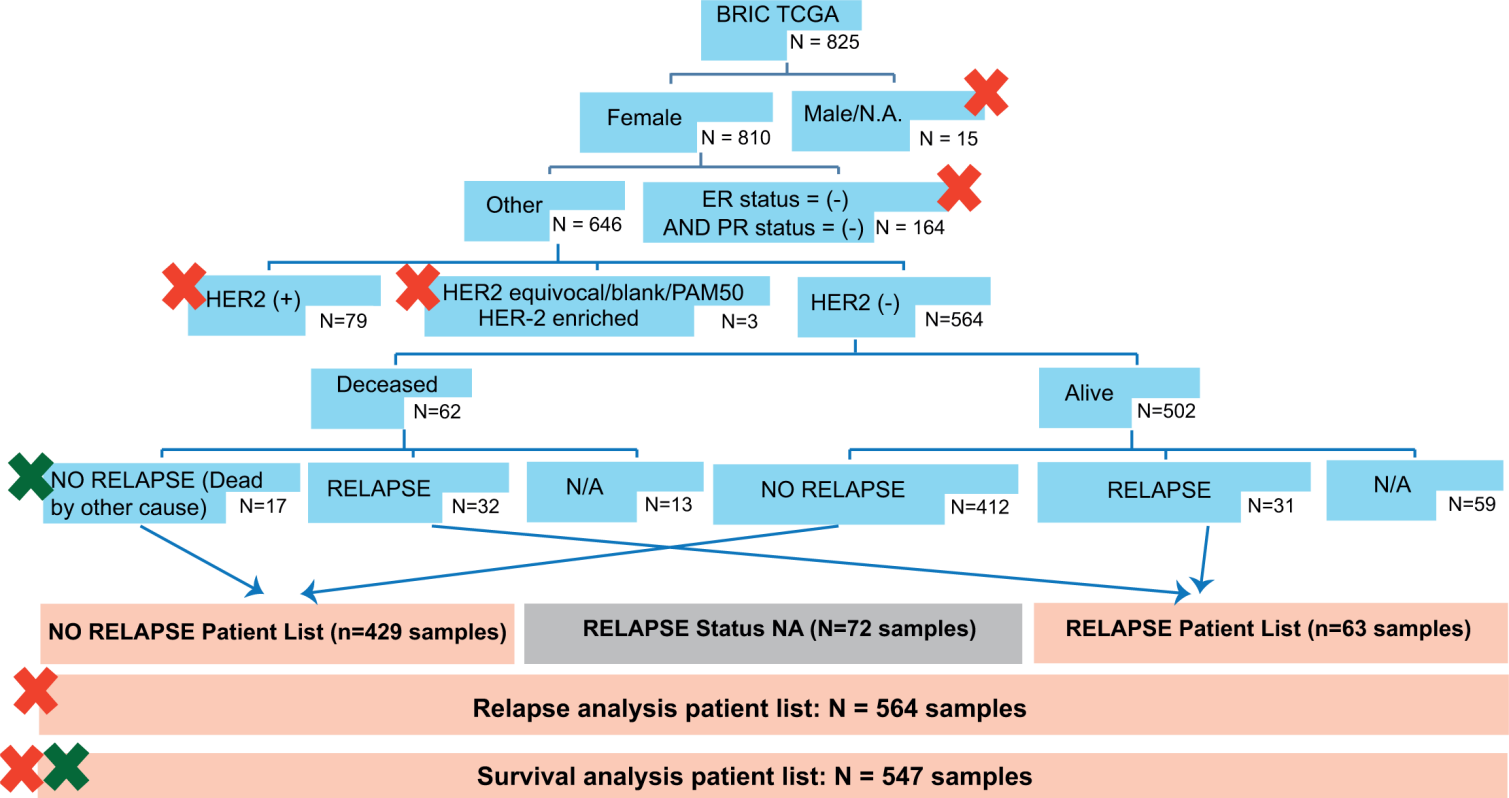
External testing—Cases valid for analysis from the TCGA series. Male or nonreported patients were excluded; patients negative for both hormone receptors and/or HER-2–positive, equivocal, unavailable, and/or falling in the HER-2–enriched cluster were excluded as well. We excluded from the relapse analysis patients without a follow-up status data; in addition, 17 additional patients were excluded from the survival analysis due to death by a non–tumor-related cause (non-relapsed).

The diagram shown in Fig 2 shows the HRPBC cases eligible for external validation among the TCGA series[8, 9].

The relative frequency of tumor-specific alterations in the candidate genes that had deleterious consequences (mutations, amplifications/deletions, mRNA up- or down-regulation) was determined in the TCGA relapsed versus nonrelapsed cases (Table 3). In addition, a log-rank test for overall survival was performed gene by gene, comparing patients with any somatic alteration in the gene versus those patients with no alterations (Table 3). Whether there was an association between the presence of alterations in the tested genes and disease relapse is included in S4 Table.

**Table 3:** Comparative Frequency of Candidate Genes in Relapsed Versus Nonrelapsed HRPBC CGA Cases.

Gene	All cases (n = 564)	Non-relapsed (n = 429)	Relapsed (n = 63)	P value*	Log-rank** (n = 547)
*PIK3CA*	28.5%	30.3%	25.4%	0.424	0.69
*TP53*	15.2%	14.2%	20.6%	0.184	0.88
*MYC*	11.9%	9.8%	19%	**0.027**	**0.05**
*CDH1*	9.2%	10.5%	4.8%	0.153	0.32
*KMT2C*	9.2%	8.4%	14.3%	0.129	0.21
*GATA3*	8.3%	8.4%	7.9%	0.904	0.61
*NCOR1*	7.8%	7.2%	9.5%	0.516	0.76
*PTEN*	6.6%	6.3%	14.3%	**0.023**	0.77
*ARID1B*	6.6%	7.5%	3.2%	0.211	0.12
*LAMB3*	6.4%	5.6%	4.8%	0.787	0.12
*DNAH5*	5.9%	6.5%	0%	**0.037**	0.17
*KMT2D*	5.5%	6.1%	7.9%	0.569	0.54
*LAMA4*	4.3%	4.2%	1.6%	0.317	0.89
*MYO3A*	4.1%	4.2%	4.8%	0.834	0.74
*CSF1R*	3.5%	3.5%	7.9%	0.095	0.18
*MYB*	3.0%	3.0%	4.8%	0.472	0.69
*EPHA7*	2.0%	1.4%	3.2%	0.298	**0.01**
*KDM6A*	0.9%	1.2%	0%	0.390	0.47
*KDM5C*	0.4%	0.5%	0%	0.589	0.96
*FOXO4*	0.2%	0.2%	0%	0.704	N/A

The genetic alteration percentages in the relapsing versus nonrelapsing patients are compared in 564 patients, for whom tumor-related relapse information was available. However, the survival information (log-rank) is obtained from a population of 547 patients because 17 patients died from a cause unrelated to the tumor, what could bias the study between genetic alterations and survival; these 17 patients were excluded from the analysis.

* Two-tailed Z-test for the comparison of the percentage of altered cases of each gene on the two populations: relapsed *versus* nonrelapsed patients. An alternative testing (Fisher's exact test, interrogating whether there is a significant association between having mutations in the genes under study and relapsing, is included in S4 Table. The P-values are similar to those obtained for the Z-test and do not alter the genes selected for signature building.

**Log-rank test for overall survival comparing the outcome of patients with deleterious alterations in each of the query genes versus the rest of the cohort.

**Fig 3:**
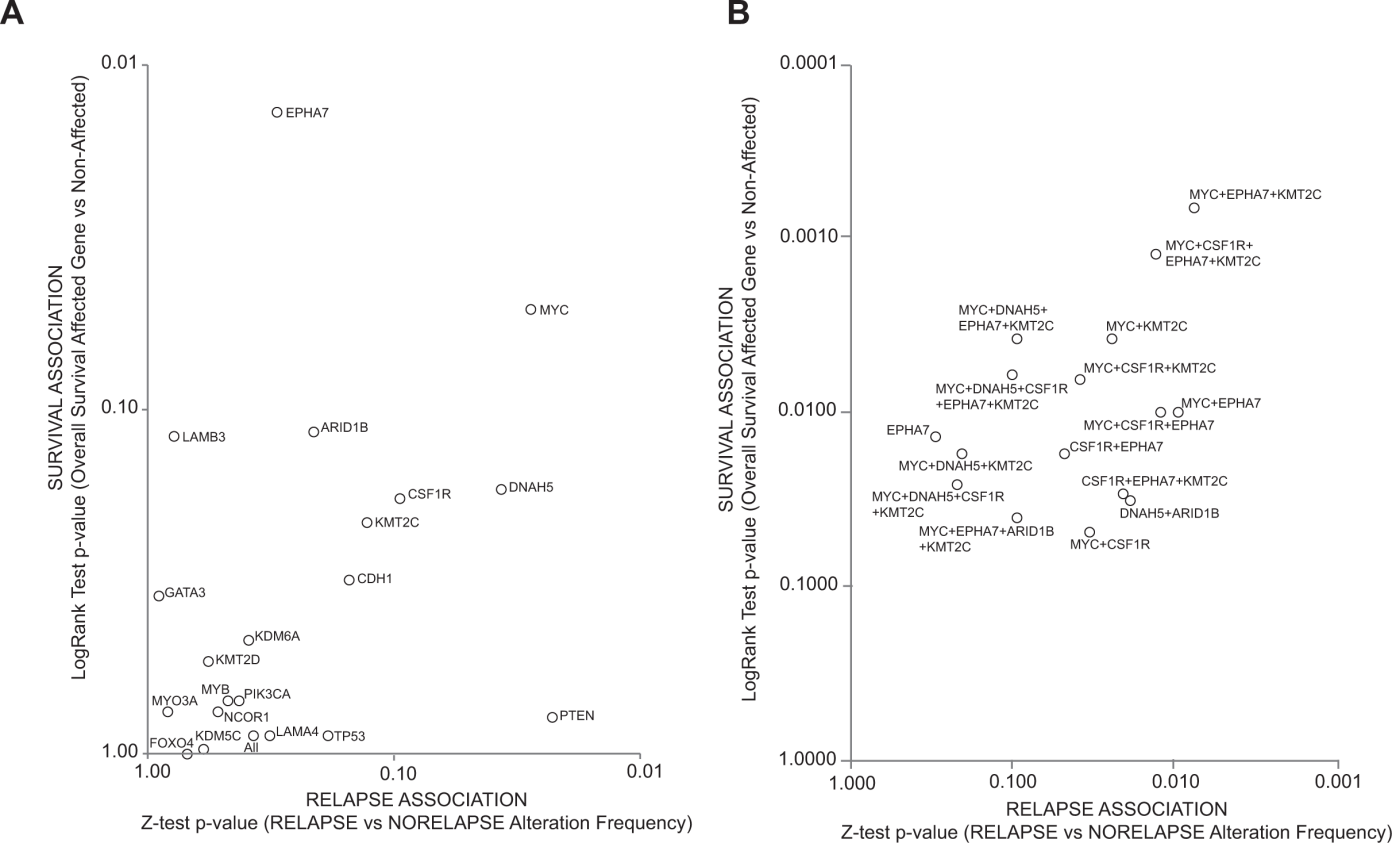
Candidate genes and signatures for prognostic testing. **(A)** Candidate genes ranked according to their association with relapse (X axis) and survival (Y axis) in the TCGA series. A smaller p-value in both axes (top-right of the scatterplot) indicates greater association between the query gene and the associated variables. **(B)** Candidate signatures ranked as in (A). Only the signatures with overall survival log-rank test p<0.05 are represented.

We then aimed to generate the most parsimonious signature of genes that could segregate the disease course based on their alterations. We plotted the z-test p-value of the proportion of altered cases in relapsed versus nonrelapsed populations against the p-value yielded by the log-rank test for each gene (Fig 3A). Theoretically, genes falling within the upper/right-side areas would be those with a higher chance of being included in the final model. We then combined the top six genes (*MYC*, *DNAH5*, *CSFR1*, *EPHA7*, *ARID1B*, and *KMT2C*) in various signatures (containing 3, 4, 5 or 6 genes) and each resulting signature was tested in the same way. The gene signatures with significant (p<0.05) overall survival in altered *versus* nonaltered cases are shown in Fig 3B.

**Fig 4:**
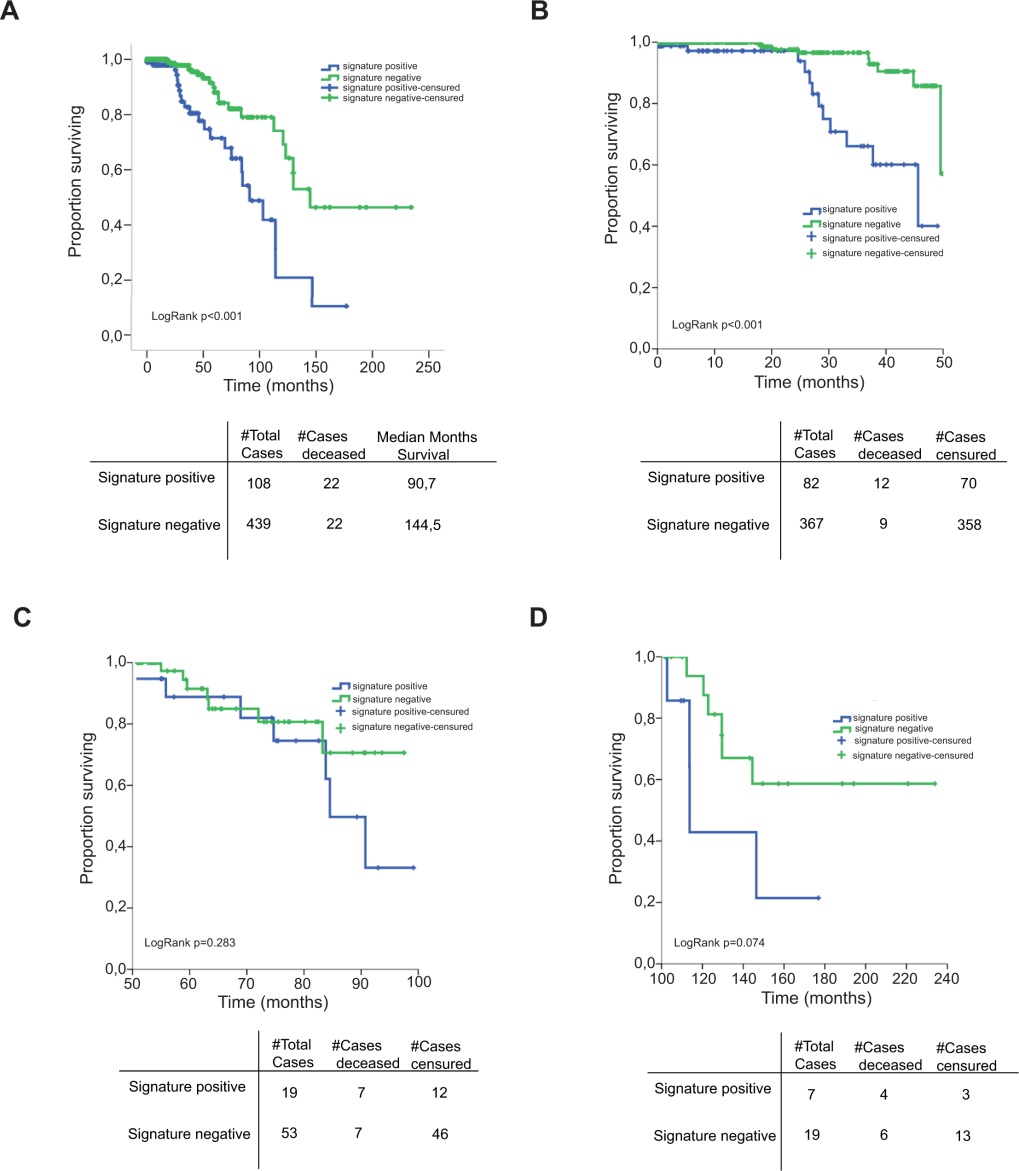
Kaplan-meier curves for patients positive versus negative for the signature. **(A)** Overall survival segregating patients by being positive (alteration in either MYC, EPHA7 and/or KMT2C) or negative (lack of alterations in the three genes) for the most parsimonious gene signature, showing the complete follow-up (250 months) of the TCGA HRPBC cohort. Log-rank test p<0.001. **(B)** Overall survival of the TCGA HRPBC cohort split by the positivity or negative of the three-gene signature during the first 50 months (log-rank p<0.001), months 50 to 100 (log-rank test p<0.283) **(C)** and beyond 100 months (log-rank p = 0.074) **(D)**. It can be observed how the majority of the deaths in the three-gene positive patients occur during the first 50 months, when they are receiving adjuvant hormonal treatment. "Signature-positive" means having an alteration with functional impact (mutation, amplification/deletion, mRNA expression up- or down-regulation) in either of the three genes according to the TCGA data; "Signature-negative" means lack of alterations in the three genes.

Among the combinations, the one found in patients with a higher difference in overall survival was also the most parsimonious (Fig 4A). Patients with alterations in *MYC*, *KMT2C*, and/or *EPHA7* had a median overall survival 4.5 years shorter than the remainder [90.7 months *versus* 144.5 (p = 0.000103)]. There were 45 deaths in the TCGA series. The first group (signature positive) was constituted by 108 patients, whereas the second (patients without any alteration in those three genes) was constituted by 439. Half of the deaths (n = 22) registered in the TCGA cohort were identified by the signature. The hazard ratio of death was increased by 3.25-fold by the presence of alterations in any of the three genes (p<0.0001). If adjusted by other variables known to influence the disease outcome that were registered in the TCGA database (age, T- and N-stage), the value of the signature was preserved (328% increase in the risk of death for the patients positive versus negative for the signature; S5 Table). Only 5% of the patients with negative signature died during a 250-month follow-up interval. Interestingly, the deaths in the signature-positive group started occurring early during the hormonal treatment: half of the events occurred between 2 and 4 years of follow-up; however, the increased relapse rate continued until the end of the follow-up (Fig 4B, 4C and 4D). Thus, taken together, these data suggest a relation between the three-gene signature positivity and early failure to hormonal treatment.

### *KMT2C*, *EPHA7*, and hormone resistance

We next sequenced 11 HRPBC cell lines (KPL1, EVSAT, ZR75-1, CAMA1, HCC1428, HCC1500, MCF7, T47D, MDA-MB175-VII, BT483, and MDA-MB-415) to study the role of *KMT2C* and *EPHA7* in the adverse course of HRPBC; the role of *MYC* has been extensively assessed elsewhere [30–34].

**Fig 5:**
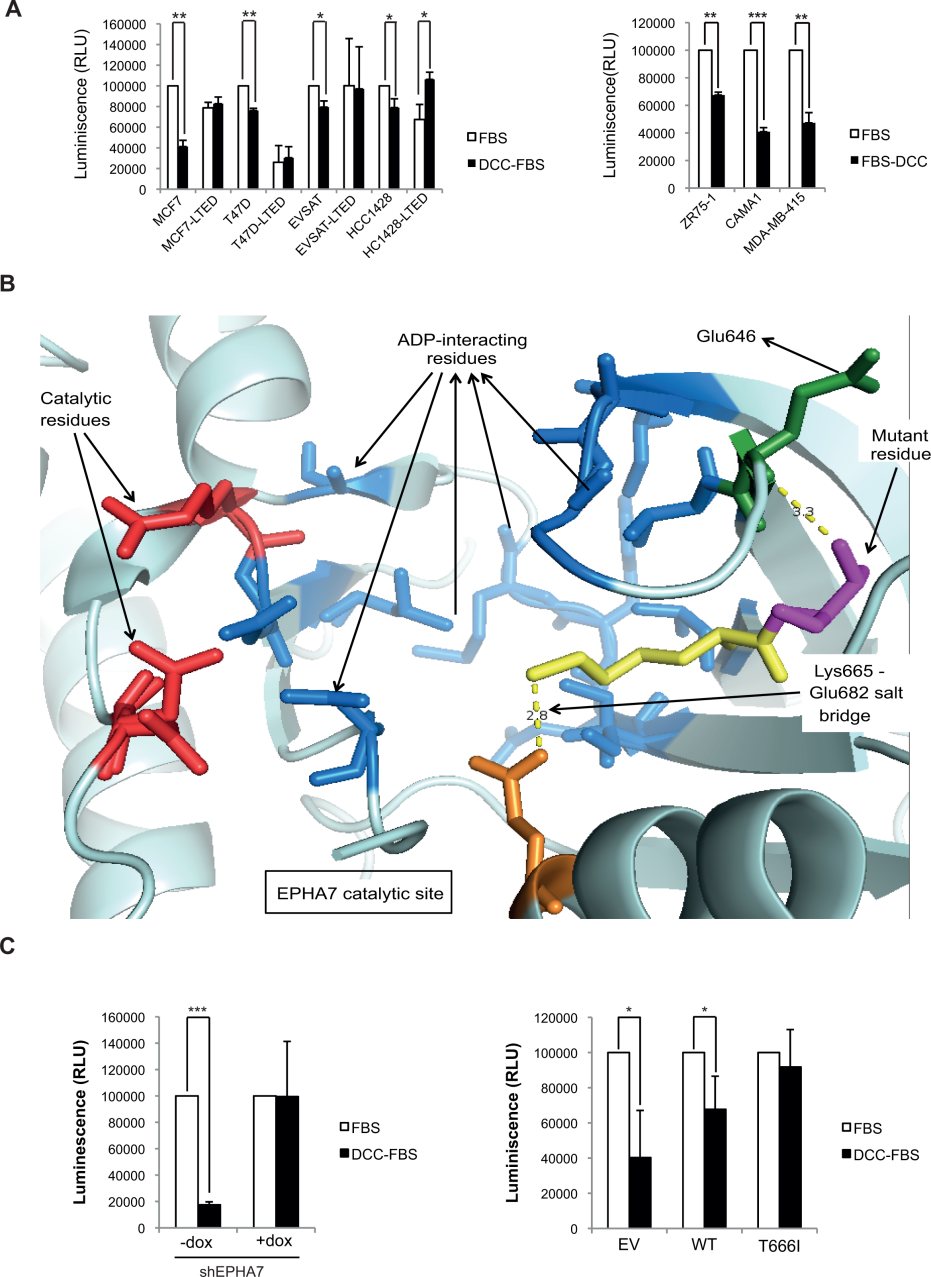
KMT2C and EPHA7 mutations are related with hormonal resistance. **(A)** Left panel: Cell growth (luminescence units, normalized to the growth of the parental clone in complete medium) in complete medium (FBS) or after 96 hours of estrogen deprivation (DCC-FBS). For each cell line, the parental clone and the clone isolated after 1 year of culture maintenance in DCC FBS are shown. Right panel: baseline response to estrogen withdrawal for non-*KMT2C*-mutant cells. **(B)** Three-dimensional structure of the kinase domain of EPHA7. The relevant residues are represented in “stick”-format. The catalytic residues are represented in red; the ADP-interacting residues, in cyan; and residue 666 is in purple. In the wild-type protein lysine-665 next to residue 666 makes a salt bridge with the side chain of glutamate-682 that coordinates the beta-phosphate of ADP/ATP. The side chain of wild-type threonine-666 is in direct contact with the main chain of glutamate-646. This region is highly conserved among tyrosine kinases; in particular, the threonine residue is conserved in all ephrin receptors (except 8 and 10). The mutation to isoleucine in 666 would change the hydrophobicity of that residue and would mean that it moves away from glutamate-646. This is likely to lead to a change in the orientation of lysine-665, which in turn may break the salt bridge, affect the coordination of the ADP/ATP, and impair the catalytic activity. **(C)** Left panel: CAMA1 cells were stably infected with a TRIPZ lentiviral vector carrying an inducible shRNAmir against *EPHA7* gene. shEPHA7 induction restored cell growth inhibition induced by DCC (82.5%). Right panel: CAMA1 cells transiently transfected with empty vector (EV), WT- or T666I-mutant EPHA7-containing vector. The growth arrest was rescued by T666I vector (8.2%) but not the WT vector (32.3%) compared with EV (59.7%). Luminiscence values normalized as in **(A)**. *p<0.05, **p<0.005, ***p<0.001.

MCF7, EVSAT, HCC1428, and T47D harbored the D348N mutation in *KMT2C*. As virtually all HRPBC patients receive prolonged hormone treatment, and adverse disease course is related to failure to hormone treatment, we cultured the cell lines for >1 year in LTED conditions to determine whether they would acquire an estrogen-independent phenotype [28]. The four cell lines with the mutation in position 348 of the *KMT2C* gene (T47D, MCF7, HCC1428, and EVSAT) acquired the LTED-R phenotype; i.e., after 1 year in culture in DCC FBS, they were insensitive [evidenced by the lack of growth arrest (or even increased growth in absence of estradiol) compared to the parental clones, which showed a growth arrest of 58,7% (MCF7), 23,9% (T47), 20,5% (EVSAT) and 21% (HCC1428) when deprived of estradiol] to temporary estrogen withdrawal (Fig 5A, left panel). The remainder cell lines stayed in either LTED-Q for >70 weeks (ZR75-1, MDA-MB-415, KPL1, and MDA-MB-175-VII) or simply failed to be cultured in DCC (CAMA1, HCC1500, and BT483). The behavior of several of these cell lines is shown in the right panel of Fig 5A. The growth arrest of ZR75-1 (32,5%), CAMA1 (59,1%) and MDA-MB-415 (52,7%) in response to estradiol withdrawal was similar to the baseline growth arrest observed in the *KMT2C*-mutant ones. However, we were unable to recover viable clones upon LTED in any case. These data suggest that the D348N mutation elicits early escape from hormone treatment.

Ephrin receptor A7 is a membrane-bound receptor tyrosine-kinase (RTK) protein. One study showed a tumor-suppressor role of EPHA7 in follicular lymphoma [35]. We found two tumors in our series with the T666I mutation; this change is likely to destabilize the binding of ATP and therefore decrease the catalytic activity of the kinase domain (Fig 5B). Mutations of a similar nature as well as mRNA down-regulation were found in the TCGA series. Thus, we generated both stable cell lines expressing a shRNA against *EPHA7* and transient overexpression of *EPHA7* wild type and T666I mutant proteins (no cell line from our panel had a mutant *EPHA7* variant). Among the cells without *KMT2C* mutation with detectable EPHA7 expression, we only achieved stable >75% downregulation of EPHA7 mRNA upon doxycycline induction in the CAMA1 cell line. This led to a complete reversion of the strong cell-growth inhibitory effect exerted by estradiol deprivation (Fig 5C). CAMA1 cells transfected with empty vector, or overexpressing the wild-type *EPHA7* gene, showed as well a strong inhibitory response to estradiol withdrawal; this response was reverted with the expression of the T666I mutant variant (Fig 5C). Taken together, these data suggest that a decrease in EPHA7 catalytic function (mediated through mRNA downregulation or kinase-domain mutations) contributes to the escape against hormone blockade.

## Discussion

Hormonal treatment is the backbone of the clinical management of HRPBC. A percentage of patients are initially refractory, but the majority remains sensitive for many years. When one hormonal agent stops being effective, switching to a different hormonal agent still controls the disease. The loss of hormonal sensitivity means an inflection point in the evolution of HRPBC patients and is associated to an adverse prognosis. Novel drugs, directed against MTOR[2] and CDK4/6[3] have helped to delay the acquisition of hormonal resistance and have meant important advances in terms of overall survival. However, the acquisition of resistance is multifactorial, and on top of the identified mechanisms, others remain to be identified[1].

Similarly to other malignancies, NGS studies can help to uncover genes or pathways related to hormonal resistance. One study sequenced exclusively treatment-naive HRPBC patients exposed to aromatase inhibitors in monotherapy prior to surgery[16]. This study found, using a pathway-oriented approach, that many pathways were altered in an overlapped manner between responders and non-responders but DNA replication, mismatch repair and TP53 signaling pathway were enriched in the non-responders We sequenced a small set of paired primary-metastatic tumors exposed long-term to hormonal blockade that experienced distant relapse, searching for novel genes involved in response/resistance to hormone treatments or accounting for disease relapse. Since genetic variants present in clones with small representation in the primary tumors can be missed if low coverage is applied, we sequenced our tumors at ultra-high depth in order to find genetic variants that were present in a small percentage of tumor cells in the primaries but expanded their representation in the metastases; such variants would be excellent candidates for being novel drivers of hormonal resistance. Unfortunately, we did not find any variant that showed significant clonal expansion repeatedly across several pairs (Table 2). In addition, the CGH arrays did not pinpoint any region that increased its copy number in the metastatic tumors versus the primaries (data not shown). Because of the small number of tumor pairs, we can not conclude that amplification or clonal expansion from the primary to the metastatic lesion does not account for hormonal resistance; with our design we would have been able only to detect potential genetic "hits" (expanded or amplified) to validate externally. Alternatively, new mutations not present in the primary tumor arising during the cancer natural history may account for the metastases and may be present only in the distant lesions—such variants might be detectable only by whole-exome sequencing, since it is likely that most of those variants would occur in regions outside of our panel. Since we did not identify any hits with our panel-strategy, we focused in the variants that were present in more than one tumor, under the assumption that the tumors that experience relapse (i.e., our series) would be genetically enriched for the variants accounting for hormonal resistance compared to incident cases (i.e., TCGA or others).

Interestingly, our pairs showed a high stability from the primary to the metastatic lesions. All tumors were more similar to their pair than to any other (Fig 1). Few variants (or copy-number variations) were present in one tumor of the pair and absent in the other (Table 1, S2 and S3 Tables). Several variants were recurrently mutated (Table 1) or amplified in the CGH arrays (S2 Table), and thus we selected them for building prognostic signatures within the TCGA HRPBC cases, in order to detect variants associated with shorter survival. In this setting, shorter survival could be a non-specific marker of hormonal resistance, since HRPBC patients are exposed long-term to these therapies during their natural history.

Although building prognostic signatures was not our primary objective, our study uncovered prognostic associations for several genes and survival in HRPBC. Several genes were more frequently mutated in the relapsing versus nonrelapsing cases or associated with worse survival in the TCGA HRPBC series (Table 3). By combining them into 3-, 4-, 5- or 6-gene signatures (Fig 3A and 3B) we found that patients showing alterations (mutations, copy number alterations, or mRNA up/downregulation) in either MYC, EPHA7 and/or KMT2C experienced a much worse outcome than the remainder (Fig 4A). The fact that many of the patients with alterations in those genes ("signature positive") relapsed and died early during disease course (Fig 4B, less than 5 years) is highly indicative of hormonal failure. HRPBC patients are commonly prescribed a minimum of 5 years of adjuvant hormonal treatment; relapse during the first 5 years is uncommon; however, many patients positive for the signature experienced disease relapse and death quite early, suggesting an implication of the genes in the signature in hormonal resistance. The increased risk of death persisted for those patients until 20 years of follow-up. It is important to mention that the TCGA data allow interrogation of alterations in several "tiers" of information. Our series only gathered data from genetic mutations recurrently present in relapsed cases (three more genes were identified by CGH—KDM5C, KDM6A and FOXO4—but did not show association in the external testing). However, the TCGA series interrogated gain or loss of function for every gene by different mechanisms, such as mutations, copy number alterations or mRNA up or down-regulation. The function of a genetic product can be altered by any of those mechanisms, and interrogating all of them allows detecting more cases where the function of a specific gene is affected. One hundred and eight cases of the HRPBC cases of the TCGA series harbored alterations of any type in MYC, KMT2C or EPHA7 and showed adverse disease course, what led us to explore their association with hormonal failure *in vitro*. The signature preserved independent prognostic value when adjusted by T, N and age. However, this should be interpreted with caution, since some of the factors implicated in the disease course, such as grade, Ki67, and most importantly, the type of treatments administered after relapse, were not gathered in most of the TCGA cases.

Finally, it has been recently shown that mutations in the *ESR1* gene, which encodes for the estrogen receptor (ER), arise as a result of chronic exposure against hormonal blockade (during the adjuvant or the metastatic setting)[13–15]; these mutations are virtually undetectable in primary tumors[5, 8–12, 16]. These mutations lead to hyperactivation of the ER signaling system even in absence of estrogens[13–15] and are linked to an adverse disease course[13, 36–39]. Our gene panel was designed before these mutations were first reported and thus *ESR1* was not sequenced in our study. We tested the prognostic role of *ESR1* mutations in the TCGA series but, as expected, we did not find any case that harbored the mutations described to activate ER. We found two mutations [one frameshift deletion (pP29Sfs*79) in one relapsing patient and one base-pair substitution (pP222S) in one non-relapsing patient; the first predicted to produce a non-functional protein and the second of uncertain signification] in the TCGA series, but none of them was theoretically associated with increased ER function. Despite the value of *ESR1*-activating mutations in the advanced disease role, it is unlikely that such mutations can be included in prognostic scores or models based on primary tumors assessment.

The main objective of our study was to detect novel genes involved in hormonal resistance, since for biomarker purposes external (and ideally prospective) validation of the candidate genes in the signature is required. The variants in the signature with the most significant prognosis association appeared to be genes functionally associated with hormone resistance. The role of MYC in hormonal resistance is already known [30–34]; however, to our knowledge, EPHA7 and KMT2C have not yet been associated with it. KMT2C is large protein (530 kDa). It contains a SET domain capable of methylating lysine 4 on histone H3K4 (a marker associated with active transcription)[40]; it can also associate with other proteins and form the histone H3 demethylase UTX complex, which demethylates H3K27 (active transcription) [41]. The D348N mutation affects one of the zinc-finger domains of the protein, and thus it may affect the expression levels of many genes [29]. Since the cell lines that harbored the D348N mutation in *KMT2C* in our study were able to acquire an estrogen-independent phenotype, but the other lines without the mutation could not (Fig 5A), makes *KMT2C* a strong candidate for being a novel driver of hormone resistance. The zinc finger harboring the mutation is a mutational hotspot according to the COSMIC database [42]. According to the latest updated ICGC data, *KMT2C* is the sixth most frequently affected gene across 43 distinct cancer genomes when only variants with severe impact are considered (S1 Fig). Other studies support epigenetics as a mediator of hormonal resistance [43, 44]. Along a similar line of thought, *EPHA7*, when inactivated by mutations in the kinase domain or down-regulated, seems to be related to hormone resistance (Fig 5B and 5C). Taken together, our study proposes *KMT2C* and *EPHA7* as novel drivers of hormone resistance.

The strengths of our study are the detection of rare variants by ultra-deep sequencing and the subsequent *in vitro* preliminary experimentation. The main weaknesses are the low number of pairs analyzed (although we did not attempt to identify *all* variants associated to hormonal resistance, but simply identify new ones) and the fact that we only sequenced genes mutated in at least 1% of breast cancers. It is possible that genetic regions that we did not sequence experienced changes from the primary to the metastatic tumors that account for hormonal resistance (although our CGH data, which are genome-wide, suggest otherwise; however, with the low number of patients is not possible to draw conclusions). One of the first studies assessing the evolution of the genome during the natural history of the tumors identified thousands of novel base-pair changes in the metastatic lesions[45]. The fact that we did not find gross alterations in the metastases versus the primary with a genome-wide interrogation by CGH arrays does not preclude the occurrence of multiple novel, undetected mutations (since an important percentage of HRPBC patients belong to the "copy-number devoid" cluster)[11].

Due to the size of our series, we have likely detected only a few genetic factors involved in HRPBC relapse and hormone resistance. Both our study and other primary-metastatic pair studies cover a limited genomic range within custom or existing gene panels, and thus the emergence of novel mutations outside the studied regions cannot be excluded. The completion of large-scale studies such as the AURORA initiative[46] will likely uncover more genetic variants related to distant relapse and variants that emerge exclusively in the distant metastasis. Future genetic studies will validate whether the presence of KMT2C, EPHA7 and/or MYC alterations in animal models will determine whether these three genes cause by themselves or are able to define as biomarkers a true functional disease sub-cluster within HRPBC.

## Conclusion

HRPBC is a heterogeneous disease but is usually responsive to hormonal therapies. When patients stop responding to hormonal therapies the prognosis changes dramatically. We sought to identify novel genetic variants associated to the loss of hormonal sensitivity. By analyzing pairs of primary and metastatic tumors we identified two novel genes linked to adverse clinical course: *KMT2C* and *EPHA7*. The TCGA data suggest that patients with alterations in those genes in the primary tumor have a high risk of death, in particular early during the adjuvant hormonal treatment. *In vitro* studies suggested that mutations in those genes are related with the acquisition of resistance against hormonal blockade, the mainstay of treatment against HRPBC. *KMT2C* and *EPHA7* are thus potential novel druggable candidates that should be explored.

## Supporting Information

S1 Fig Distribution of non-synonimous SSMs (Single Somatic Mutations) across all cancer genomes in the ICGC data portal (release 19) along the protein sequence of KMT2C. 1,185 donors are affected by 1,191 mutations in *KMT2C* across 43 distinct cancer genomes, making it the 6^th^ most common cancer gene with SSMs that have a hogh functional impact. Main mutational hotspots are observable around the zinc-finger domains. Red, green and grey circles represent somatic changes with predicted high, low or no functional impact, respectively. Vertical axis represents the number of donors with the mutation. Horizontal axis indicates the protein residue number. Mutation p.D348N is highlighted with a blue Squire (dcc.icgc.org).
(EPS)

S1 Table Genes interrogated in the CNIO-BR-004 study. A custom panel covering the coding DNA sequence of the 106 genes that are known to be altered in at least 1% of the HRPBC cases was designed with SureSelect technology. Copy number alterations (CNAs) were studied by comparative genomic hybridization (CGH) using a Human Whole Genome 8x60k oligonucleotide array-CGH (Agilent Technologies), to query the 101 regions gained or lost (CNAs) in at least 1% of HRPBC cases.
(DOCX)

S2 Table Regions gained and/or lost in the pairs. A summary of the regions gained and/lost according to the CGH arrays data in each of the pairs studied.
(DOCX)

S3 Table Variants differed between the metastatic and primary lesions in each pair. List of the 1071 variants showing whether differed or not between the metastatic and primary lesions in each pair.
(XLSX)

S4 Table Fisher's exact test by gene (association with relapse). To test whether there was an association between the presence of alterations in the tested genes and disease relapse, we have computed all the Fisher's exact test P-values for Table 3.
(DOCX)

S5 Table Cox's proportionate hazards model. Cox multivariate model, adjusting the presence or absence of the signature *MYC*, *KMT2C*, and/or *EPHA7* by other variables known to influence the disease outcome that were registered in the TCGA database (age, T- and N-stage). The model was significant (Chi-square 33.1, P<0.001).
(DOCX)
