# Supplementary material for: Analysis of Paired Primary-Metastatic Hormone-Receptor Positive Breast Tumors (HRPBC) Uncovers Potential Novel Drivers of Hormonal Resistance
Source: PLoS One. 2016 May 19;11(5):e0155840. doi: 10.1371/journal.pone.0155840 (PMC4873174; doi:10.1371/journal.pone.0155840)
Supplement: S1 Table — A custom panel covering the coding DNA sequence of the 106 genes that are known to be altered in at least 1% of the HRPBC cases was designed with SureSelect technology. Copy number alterations (CNAs) were studied by comparative genomic hybridization (CGH) using a Human Whole Genome 8x60k oligonucleotide array-CGH (Agilent Technologies), to query the 101 regions gained or lost (CNAs) in at least 1% of HRPBC cases. (DOCX) [file pone.0155840.s002.docx]

**S1Table**: **Genes interrogated in the CNIO-BR-004 study**

| **Genes mutated in breast cancer** | **Genes mutated and/or experiencing CNAs in breast cancer** | **Genes experiencing CNAS in breast cancer** |
| --- | --- | --- |
| IDH1 | PPP2R2A | CDKN2A |
| TP53 | MTAP | CDKN2B |
| USH2A | MAP2K4 | T cell receptor (TCR) loci on chromosomes 7 (TRG) and 14 (TRA) |
| MYO3A | PTEN | ZNF703 |
| ATR | ERBB2 | CCND1 |
| CDH1 | MYC | CDKN2AIP |
| MLL3 | CCND1 | AIM1 |
| MAP3K1 | MDM2 | PAK1 |
| CDKN1B | RPS6KB1 | RSF1 |
| TBX3 | CCNE1 | INTS4 |
| RUNX1 | MDM4 | SEPHS1 |
| LDLRAP1 | CDK3 | ZMIZ1 |
| STMN2 | CDK4 | FOXM1 |
| MYH9 | CAMK1D | SDCCAG1 |
| AGTR2 | PI4KB | IGF1R |
| SF3B1 | NCOR1 | GNRH1 |
| CBFB | EMSY | KCTD9 |
| MAP3K12 | PARK2 | CDCA2 |
| MAP4K3 | RB1 | EBF2 |
| MAP4K4 | PDGFRA | BNIP3L |
| MAPK15 | EPHA7 | PNMA2 |
| MAPK3 | CSF1R | DPYSL2 |
| APAF1 | DDR1 | ADRA1A |
| THBS1 | MET | STMN4 |
| IGF1 | KIT | TRIM35 |
| DDR2 | PIK3CA | AQP11 |
| RPS6KB1 | AKT1 | CLSN1A |
| BRAF | AKT2 | KCTD14 |
| AFF2 | MAP3K4 | THRSP |
| PIK3R1 | MAP2K3 | NDUFC2 |
| PTPN22 | GATA3 | ALG8 |
| PTPRD | CDC25 | KCTD21 |
| NF1 | FOXO3 | USP35 |
| CCND3 | CDK2 | GAB2 |
| CTCF | MYB | DNAH9 |
| ZFP36L1 | MLL2 | ZNF18 |
| GPS2 | MLL3 | c11orf67 |
| OR6A2 | MLL4 | KRAS |
| CLEC19A | MLL5 | FOXO1 |
| FAM47C | KDM6A | FOXO4 |
| PIWIL1 | KDM5B | FGFR1 |
| FOXA1 | KDM5C | FGFR2 |
| SRPR | ARID1A | FGFR3 |
| OR2L2 | ARID2 | IGF1R |
| ARID1B | ARID3B | Cyclin D1 |
| CASP8 | ARID4B | CDK6 |
| STK11 | SMAD4 | INPP4B |
| SMARCD1 | ATM | ZNF217 |
| SETD2 | ASXL1 | EGFR |
| DNAH5 | KRAS | ERBB3 |
| COL12A1 |  | ERBB4 |
| LAMA4 |  |  |
| DNAH3 |  |  |
| LAMB3 |  |  |
| BRCA1 |  |  |
| BRCA2 |  |  |
