# Supplementary material for: Analysis of Paired Primary-Metastatic Hormone-Receptor Positive Breast Tumors (HRPBC) Uncovers Potential Novel Drivers of Hormonal Resistance
Source: PLoS One. 2016 May 19;11(5):e0155840. doi: 10.1371/journal.pone.0155840 (PMC4873174; doi:10.1371/journal.pone.0155840)
Supplement: S2 Table — A summary of the regions gained and/lost according to the CGH arrays data in each of the pairs studied. (DOCX) [file pone.0155840.s003.docx]

**Supplementary table 2: regions gained and/or lost in the pairs.**

| **Pair** | **Primary / Metastatic tumor lesion** | **Genes** |
| --- | --- | --- |
| **Pair A** | Primary tumor | Gains: *FOXO4, KDM6A, KDM5C.*  Losses: *MAP2K4, CSF1R, MAP3K4, PARK2, ZNF18, DNAH9* |
|  | Metastatic tumor | Gains: None  Losses: None |
| **Pair C** | Primary tumor | Gains: *FOXO4, KDM6A, KDM5C, CSF1R*  Losses: none |
|  | Metastatic tumor | Gains: *FOXO4, KDM6A, KDM5C.*  Losses: none |
| **Pair E** | Primary tumor | Gains: *FOXO4, KDM6A, KDM5C, CSF1R, AQP11, KCDT14, PAK1, RSF1, AAMDC, INTS4, ZNF703, FGFR1*  Losses: none |
|  | Metastatic tumor | Gains: *FOXO4, KDM6A, KDM5C, PAK1, ARID4B*  Losses: none |
| **Pair J** | Primary tumor | Gains: *FOXO4, KDM6A, KDM5C.*  Losses: *MAP2K4, KMT2B, AKT2, ZNF18, DNAH9* |
|  | Metastatic tumor | Gains: *FOXO4, KDM6A, KDM5C.*  Losses: none |
| **Pair K** | Primary tumor | Gains: *FOXO4, KDM6A, KDM5C, ZNF703*  Losses*:* none |
|  | Metastatic tumor | Gains: *FOXO4, KDM6A, KDM5C.*  Losses: none |
| **Pair L** | Primary tumor | Gains: *FOXO4, KDM6A, KDM5C.*  Losses: none |
|  | Metastatic tumor | Gains: *FOXO4, KDM6A, KDM5C.*  Losses: none |
| **Pair M** | Primary tumor | Gains: *FOXO4, KDM6A, KDM5C.*  Losses: *KMT2B, AKT2, EGFR* |
|  | Metastatic tumor | Gains: *FOXO4, KDM6A, KDM5C, KMT2E, MET, KMT2C, TARP, EGFR, CDK6.*  Losses: *MAP2K3, MAP2K4, ERBB2, CDK3, RPS6KB1, CSF1R, CDC25C, PPP2R2A, MTAP, ZNF18, DNAH9, FGFR3, PNMA2, GNRH1, CDCA2, STMN4, TRIM35, KCTD9, EBF2, BNIP3L, DPYSL2, CDKN2A, CDKN2B.* |
| **Pair O** | Primary tumor | Gains: *FOXO4, KDM6A, KDM5C.*  Losses: *MAP2K4, CCNE1, KMT2B, AKT2, ZNF18, DNAH9* |
|  | Metastatic tumor | Gains: *FOXO4, KDM6A, KDM5C, PI4KB, ARID4B, CCND1, RPS6KB1, CCND1, INPP4B, FGFR1.*  Losses: *ATM, MAP2K4, ZNF18, DNAH9* |

We interrogated specifically the 101 regions encoding for the genes showing CNAs in at least 1% of the cases according to the literature (depicted in Supplementary Table 1). Most of the regions did not show gains or losses; the regions among the 101 that showed any change with regard to the reference genome are shown in this table by pair and lesion. FOXO4, KDM6A and KDM5C were gained in all the tumors but the metastatic A-tumor (although they did not show significant association in the validation set). The 101 regions remained mostly stable from the primary to the metastatic tumor, as shown in the table, with few CNAs present in the metastases that were absent in the primaries.
