# Supplementary material for: Analysis of Paired Primary-Metastatic Hormone-Receptor Positive Breast Tumors (HRPBC) Uncovers Potential Novel Drivers of Hormonal Resistance
Source: PLoS One. 2016 May 19;11(5):e0155840. doi: 10.1371/journal.pone.0155840 (PMC4873174; doi:10.1371/journal.pone.0155840)
Supplement: S4 Table — To test whether there was an association between the presence of alterations in the tested genes and disease relapse, we have computed all the Fisher's exact test P-values for Table 3. (DOCX) [file pone.0155840.s005.docx]

**Supplementary Table 4: Fisher's exact test by gene (association with relapse)**

| Gene | All cases  (n = 564) | Non-relapsed  (n = 429) | Relapsed  (n = 63) | P value (Z test) | P value (Fisher's test) |
| --- | --- | --- | --- | --- | --- |
| *PIK3CA* | 28.5% | 30.3% | 25.4% | 0.424 | 0.464 |
| *TP53* | 15.2% | 14.2% | 20.6% | 0.184 | 0.188 |
| *MYC* | 11.9% | 9.8% | 19% | **0.027** | **0.048** |
| *CDH1* | 9.2% | 10.5% | 4.8% | 0.153 | 0.178 |
| *KMT2C* | 9.2% | 8.4% | 14.3% | 0.129 | 0.156 |
| *GATA3* | 8.3% | 8.4% | 7.9% | 0.904 | 1.000 |
| *NCOR1* | 7.8% | 7.2% | 9.5% | 0.516 | 0.452 |
| *PTEN* | 6.6% | 6.3% | 14.3% | **0.023** | **0.035** |
| *ARID1B* | 6.6% | 7.5% | 3.2% | 0.211 | 0.290 |
| *LAMB3* | 6.4% | 5.6% | 4.8% | 0.787 | 1.000 |
| *DNAH5* | 5.9% | 6.5% | 0% | **0.037** | **0.037** |
| *KMT2D* | 5.5% | 6.1% | 7.9% | 0.569 | 0.577 |
| *LAMA4* | 4.3% | 4.2% | 1.6% | 0.317 | 0.491 |
| *MYO3A* | 4.1% | 4.2% | 4.8% | 0.834 | 0.742 |
| *CSF1R* | 3.5% | 3.5% | 7.9% | 0.095 | 0.159 |
| *MYB* | 3.0% | 3.0% | 4.8% | 0.472 | 0.444 |
| *EPHA7* | 2.0% | 1.4% | 3.2% | 0.298 | 0.278 |
| *KDM6A* | 0.9% | 1.2% | 0% | 0.390 | 1.000 |
| *KDM5C* | 0.4% | 0.5% | 0% | 0.589 | 1.000 |
| *FOXO4* | 0.2% | 0.2% | 0% | 0.704 | 1.000 |
