# Supplementary material for: Analysis of Paired Primary-Metastatic Hormone-Receptor Positive Breast Tumors (HRPBC) Uncovers Potential Novel Drivers of Hormonal Resistance
Source: PLoS One. 2016 May 19;11(5):e0155840. doi: 10.1371/journal.pone.0155840 (PMC4873174; doi:10.1371/journal.pone.0155840)
Supplement: S5 Table — Cox multivariate model, adjusting the presence or absence of the signature MYC, KMT2C, and/or EPHA7 by other variables known to influence the disease outcome that were registered in the TCGA database (age, T- and N-stage). The model was significant (Chi-square 33.1, P<0.001). (DOCX) [file pone.0155840.s006.docx]

**Supplementary Table 5 - Cox's proportionate hazards model**

| **Predictive factor** | **Risk** | **P-value** |  |
| --- | --- | --- | --- |
| **T*** | 0.491 | 0.001 |  |
| **N*** | 0.681 | 0.168 |  |
| **Age*** | 1.03 | 0.010 |  |
| **Signature (positive vs. negative)** | 3.28 | <0.001 |  |

*The risk (protection) is shown per each unit increase (i.e., T2 vs. T1) in the T-stage. The same applies for the N-stage, although it lacked statistical significance. The risk depicted for age applies to the risk increase per year.
